# Molecular Detection of Staphylococcal Enterotoxins and mecA Genes Products in Selected Food Samples Collected from Different Areas in Khartoum State

**DOI:** 10.1155/2021/5520573

**Published:** 2021-03-19

**Authors:** Mohammed Yahya Ahmed, Hashim Abdalbagi Ali, Babbiker Mohammed Taher Gorish, Sara Omer Ali, Eman Saif Aldein Abdalrhim, Mawada Hamza Mergani, Asmaa Abass Abd Elgadir, Somaya Khalid Mohammed, Salma Omer Ahmed, Naglaa Alsaeid Musa, Alaa Saeed Ahmed, Wafaa Mohammed Abdalla, Yousif Fadlallah Hamedelnil, Ahmed Ibrahim Hashim, Hisham N. Altayb

**Affiliations:** ^1^Department of Microbiology, Faculty of Medical Laboratory Science, Sudan University of Science and Technology, Khartoum, Sudan; ^2^Department of Microbiology, Faculty of Medical Laboratory Science, Omdurman Islamic University, Omdurman, Sudan; ^3^Department of Biochemistry, Faculty of Sciences, King Abdulaziz University, Jeddah, Saudi Arabia

## Abstract

Staphylococcal food poisoning is an intoxication that results from the consumption of improperly prepared or stored foods containing sufficient amounts of one or more preformed *S*. *aureus* enterotoxins. Nowadays, many researchers worldwide noted an emergence of resistant strains such as *Staphylococci* particularly for the antibiotic methicillin. Therefore, this study was aimed to determine the existence of *Staphylococcus aureus* and its enterotoxins, mecA genes, in selected food samples. A total of 400 selected food samples were collected from different areas in Khartoum State. The selected foods included cheese, meat products, fish, and raw milk. One hundred samples from each type of food were cultivated, and the resultant growth yielded 137 (34.25%) *S*. *aureus*, 126 (31.5%) bacteria other than *S*. *aureus*, and 137 (34.25%) yielded no growth. Eighty-four of the 137 *S*. *aureus* isolates were randomly selected and tested for the presence of mecA and enterotoxin genes. The oxacillin sensitivity test showed that 15 (11%) of 137 *S*. *aureus* isolates were oxacillin resistant. The PCR assay showed that the mecA gene was detected in 15 of 84 (17%) *S*. *aureus* isolates. Simultaneously, only 2 (2.385%) out of 84 *S*. *aureus* isolates showed an enterotoxin B gene product. There was a relatively moderate prevalence of methicillin-resistant *Staphylococcus aureus* with very low frequency of enterotoxin B gene in different kinds of selected food samples collected from Khartoum State. These findings elucidate the increased risk on public in Khartoum being affected by Staphylococcal food poisoning upon consumption of dairy or meat products prepared in unhygienic conditions that could lead to intoxication by *Staphylococcus aureus* enterotoxins.

## 1. Background

Foodborne diseases (FBD) remain as one of the greatest concerns in public health and food safety; they are caused by many pathogens that contaminate food and food products [1]. Many food sources may serve as a substrate for many microorganisms which are transmitted during harvesting, storage, food processing, and handling by multiple environmental sources such as water, soil, insects, or even by the handlers [2].

Staphylococcal food poisoning (SFP) is an intoxication that results from the consumption of improperly prepared or stored foods containing sufficient amounts of one or more preformed enterotoxins [3, 4]. A wide variety of foods support the growth of *Staphylococcus aureus* and are ideal for enterotoxin production including milk, meat, meat products, dairy products, and ready-to-eat food [5, 6].

Although *Staphylococcus aureus* may produce a large variety of enterotoxins, 95% of food poisoning outbreaks are caused by classical enterotoxins A, B, C, D, and E [7]. Since these toxic proteins are capable of tolerating high temperatures up to 100°C for several minutes, improperly cooked food contaminated with bacteria or its preformed toxins in sufficient amounts could lead to Staphylococcal food poisoning within a few hours characterized by symptoms including nausea, vomiting, and diarrhea [8]. Some strains of *Staphylococcus aureus* have the ability to resist heat and drying; hence, it can easily contaminate foods. This contamination might come from food handlers or from the environment where the bacteria multiply and release toxins in uncooked or inadequately cooked foods, especially if the foods are unrefrigerated [9]. The consumption of foods of animal origin contaminated with MRSA or MRSA preformed enterotoxins could lead to serious threats to the well-being of humans due to uncountable clinical implications [10].

Nonhygienic handling practices, working conditions, and improper storage and refrigeration can all increase the opportunity for food contamination. So, it is important to follow the standard practices in food handling such as hand washing, proper cooking, proper storage, and others to reduce or prevent food contamination [11, 12].

There is paucity of information in Sudan regarding the role *Staphylococcus aureus* in food poisoning and the presence of enterotoxins in mecA genes in common consumed foods in Khartoum State. Hence, this study was conducted to determine the prevalence of enterotoxins and mecA genes in foods commonly consumed in Khartoum State, Sudan.

## 2. Methods

### 2.1. Collection of Selected Food Samples

A total of 400 samples were collected from different areas in Khartoum State (Khartoum, Omdurman, East Nile, and Khartoum North), during 2018. The type of foods included cheese, meat products, fish, and raw milk. Each sample was aseptically collected, fifteen grams of cheese samples were collected from different retailers by using sterile container, meat samples were collected randomly from supermarkets and restaurants using disposable blades, a small piece of raw meat had been splitted and transferred to the lab in sterile containers, small pieces of fish inner tissues were collected by a sterile blade and placed in sterile plain containers, and milk samples were collected in sterile containers and stored in a refrigerator at 4°C in the microbiology laboratory until examined.

### 2.2. Isolation and Identification of Coagulase-Positive *Staphylococcus* Isolates

Meat, fish, and cheese samples were enriched in peptone water. The raw milk and the enriched peptone water samples were swabbed and inoculated in blood agar medium, mannitol salt agar medium, and MacConkey's agar medium and incubated aerobically at 37°C for 24–48 hrs. The presence of *Staphylococcus aureus* was confirmed based on colony morphology, Gram's reaction, and other biochemical tests including the catalase test, coagulase test, and DNase test.

### 2.3. Antimicrobial Susceptibility Testing of Coagulase-Positive Staphylococci

The antimicrobial susceptibility test was performed by the disk diffusion method using Mueller–Hinton agar plates (oxoid) according to [13], where 4 antimicrobial agents belonging to different classes were selected including ciprofloxacin (5 *μ*g), gentamicin (10 *μ*g), oxacillin (5 *μ*g), and vancomycin (30 *μ*g). The *S*. *aureus* ATCC 52923 control strain was used.

### 2.4. DNA Extraction

DNA was extracted by the simple boiling method, in which the extracted product was obtained from overnight isolates on nutrient agar. A loop full of bacterial colony was picked from an isolate and suspended in 300 *μ*l of sterile distilled water, and 10 *μ*l of proteinase K was added and incubated at 6°C for 60 minutes. Then, it was incubated at 100°C in a waterbath for 15 minutes, and the suspension was centrifuged at high speed (10000 rpm for 10 min). The supernatant containing the genomic DNA was transferred into a fresh sterile Eppendorf tube and stored at −20°C until to be used for PCR [14].

### 2.5. PCR Detection of 16s rRNA Gene

All samples were confirmed as *S*. *aureus* by using specific housekeeping gene primer (16s) as shown in Table 1. Negative samples were excluded. The DNA amplifications were performed from a volume of 25 *μ*L of a mixture containing 2 *μ*L Maxime PCR Premix, 0.5 *μ*L of each primer, 2 *μ*L of template DNA, and 20 *μ*L of double distilled water. The amplification conditions included three steps: initial denaturation at 94°C for 5 min; 35 successful cycles of denaturation at 94°C for 45 sec, annealing at 50°C for 45 sec, and extension at 72°C for 45 sec; and the final extension at 72°C for 7 min [12].

### 2.6. PCR Detection of Staphylococcal Enterotoxins Genes

Multiplex PCR amplification was performed using the CLASSIC K960 (UK) thermocycler. PCR amplification of Staphylococcal enterotoxins (SE) genes, namely, sea, seb, sec, sed, and see, was performed using the Maxime PCR PreMix kit (iNtRON, Korea) and specific primers listed in Table 2. The PCR assay was carried out in a total volume of 25 *μ*L of mixture containing 2 *μ*L Maxime PCR PreMix, 0.5 *μ*L of each of the toxic gene-specific primers (5 *μ*L), 2 *μ*L of template DNA, and 16 *μ*L of double distilled water. The amplification conditions included three steps: initial denaturation at 94°C for 5 min; 35 successful cycles of denaturation at 94°C for 45 sec, annealing at 50°C for 45 sec, and extension at 72°C for 45 sec; and the final extension at 72°C for 7 min [12].

### 2.7. PCR Detection of mecA Gene

Primers were used for the detection of mecA gene, as shown in Table 1. DNA amplification was performed using the Maxime PCR PreMix kit (iNtRON, Korea). The PCR assay was carried out in a total volume of 20 *μ*L of mixture containing 2 *μ*L Maxime PCR PreMix, 0.5 *μ*L of each of the gene-specific primers (5 *μ*L), 2 *μ*L of template DNA, and 13 *μ*L of double distilled water. The amplification conditions included three steps: initial denaturation at 94°C for 5 min; 35 successful cycles of denaturation at 94°C for 45 sec, annealing at 52°C for 45 sec, and extension at 72°C for 45 sec; and the final extension at 72°C for 7 min.

### 2.8. Quality Control

All samples were aseptically collected and analyzed; positive control which was a well-known enterotoxin and mecA gene producing *Staphylococcus aureus* and negative control which was sterile distilled water were included during PCR running.

## 3. Results

### 3.1. Prevalence of *S*. *aureus* Isolates in Food Samples

**Figure 1:**
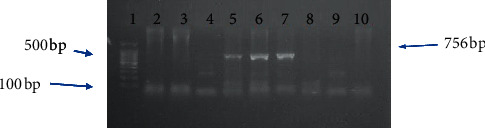
PCR amplification of 16S rRNA gene of *S*. *aureus* on 2% agarose gel electrophoresis. Lane 1 DNA ladder, MW 100–1500 bp fragments. Lanes 5, 6, and 7 show a typical band size of 756 bp corresponding to 16S rRNA of positive control isolates (isolates IDs 13, 55, and 63, respectively). Lanes 2, 3, 4, and 8 are the negative samples.

The presence of *S*. *aureus* was observed in 137 (34.25%) of the 400 food samples collected from different areas in Khartoum State. However, 126 (31.5%) of the 400 samples were identified as bacteria other than *S*. *aureus*, and 137 (34.25%) samples did not yield any growth (Table 3). All isolated *S*. *aureus* were confirmed by detection of the 16S rRNA housekeeping gene product which corresponds to 756 bp band size (Figure 1).

### 3.2. Detection of mecA and Enterotoxins Genes among *S*. *aureus* Isolates

**Figure 2:**
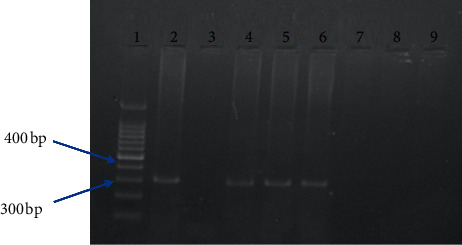
PCR amplification of mecA gene of *S*. *aureus* on 2% agarose gel electrophoresis. Lane 1 DNA ladder, MW 100–1500 bp fragments. Lanes 2, 4, 5, and 6 show a typical band size of 310 bp corresponding to mecA gene products of *S*. *aureus* isolated from samples number 13, 55, 63, and 81. Lanes 3, 7, 8, and 9 are the negative samples.

**Figure 3:**
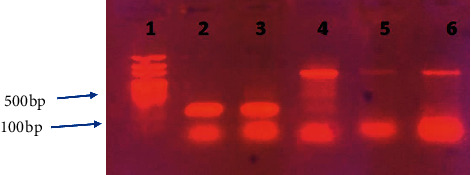
Agarose gel electrophoresis for PCR product of enterotoxin B gene (165 bp) and 16s rRNA (756 bp). Lane 1, DNA ladder 50 bp. Lane 2 shows a typical band of the positive control *S*. *aureus* enterotoxin B seb gene. Lane 3 shows a positive enterotoxin B gene product of *S*. *aureus* isolated from cheese samples (sample 16). Bands 4, 5, and 6 show a positive sample for 16s rRNA gene (isolates 16, 17, and 31).

The 84 different *S*. *aureus* isolates were randomly selected from a total of 137 *S*. *aureus* isolates. The selected isolates were further examined for the presence mecA and enterotoxin genes using specific primer in a conventional PCR assay. The mecA gene was detected in 15 (17%) *S*. *aureus* isolates (in samples number 6, 13, 55, 63, and 81 of meat; samples number 2, 11, 47, and 72 for cheese; in samples number 19, 28, 34, and 49 of milk; and in samples number 9 and 38 of fish (Table 4) (Figures 1 and 2)). However, only 2 (2.385%) of 84 *S. aureus* isolates showed an enterotoxin B gene product (both isolates were from cheese samples and the samples ID were 16 and 31), while the rest of 82 isolates were negative. All isolates were negative for other enterotoxins gene (other than seb gene) (Table 4) (Figure 3).

### 3.3. Meat Isolates' Antimicrobial Susceptibility Characteristics

Presence of *S*. *aureus* was observed in 30 (30%) of the 100 milk samples (Table 5), of which 11 (36.7%) isolates were detected in raw beef and 19 (63.3%) were identified in restaurants meat. However, 20 (20%) samples showed the growth of bacteria other than *S*. *aureus*, and 50 (50%) samples showed no growth. All tested meat *S*. *aureus* isolates were susceptible to ciprofloxacin. The resistant rates of meat *S*. *aureus* isolates to gentamicin was 4 (13.3%), and it was higher than that identified to the antibiotics oxacillin and vancomycin with 2 (6.6%) and 5 (16.7%), respectively (Table 1). The PCR assay for enterotoxin gene products showed that none of the meat *S*. *aureus* isolates produced enterotoxins genes products (Figure 3).

### 3.4. Cheese Isolates' Antimicrobial Susceptibility Characteristics

The examination of 100 cheese samples collected from different areas in Khartoum State revealed that the occurrence of *S*. *aureus* isolate was 20 (20%), while bacteria other than *S*. *aureus* represented 4%. However, none of the rest 76 (76%) showed any growth on the agar plate surface (Table 5). We found that all isolates were susceptible to both gentamicin and ciprofloxacin antibiotics. However, vancomycin showed growth-inhibition zones with 17 (85%) isolates out of 20 positive samples. Only one (5%) cheese *S*. *aureus* isolate was resistant to oxacillin (Table 1). Enterotoxin gene B (seb) was detected only in 2 (10%) of cheese isolates (samples 16 and 31), while none of other types of enterotoxin genes products was detected among these isolates (Figure 3).

### 3.5. Antimicrobial Profile for Fish *S*. *aureus* Isolates

In this study, a total of 100 fish samples (50 salted fish and 50 raw fish) were analyzed for the presence of bacterial pathogens. The study revealed that 24/100 (24%) of the fish samples had *S*. *aureus* contamination (Table 5). Antibiotic susceptibility of *S*. *aureus* was tested using the agar disc diffusion method. The results have shown that all fish originated *S. aureus* isolates were susceptible to both ciprofloxacin and gentamicin antibiotics. In contrast, the resistance rate was only 8% to both vancomycin and oxacillin antibiotics (Table 1). No enterotoxin gene product was detected during the gel electrophoresis procedure which was applied after successful cycles of conventional PCR (Figure 3).

### 3.6. Antimicrobial Profile for Milk *S*. *aureus* Isolates

Out of 100 milk samples collected from different areas in Khartoum State, 63 (63%) were identified as *S*. *aureus*, 26 (26%) were identified as bacteria others than *S*. *aureus*, and 11 (11%) showed no growth (Table 5). The antimicrobial susceptibility test was performed to all *S*. *aureus* isolates, and the result showed that the highest susceptibility rate was recorded to ciprofloxacin and gentamicin with a percentage of 98.4% (62/63 isolates) and 87.3% (55/63 isolates), respectively, followed by oxacillin with a percentage of 84% (53/63). However, the least potent antibiotic was vancomycin with a percentage of 65% (41/63) (Table 1). The enterotoxin genes results reveal that no *S*. *aureus* isolate produces such genes (Figure 3).

## 4. Discussion

In this study, the prevalence of *S*. *aureus* and MRSA and enterotoxin gene products were investigated in various food samples collected from Khartoum State markets (400 samples of milk, cheese, fish, and meat). Identification of the bacteria isolated from the selected foods through conventional methods yielded a total of 137 (34.3%) *S*. *aureus* isolates. Similar reports on foods contaminated with *S*. *aureus* from Italy and India revealed much lower percentage yielding 17.1% [15] and 12.01% [16], respectively. The previous studies conducted to detect *S*. *aureus* in various foods revealed that the contamination levels with *S*. *aureus* reported were lower than those obtained in this study. On the other hand, a study in Greece reported 47.8% of northcentral and northeastern Greece foods, which have a much higher contamination level compared to those reported in this study [3]. Those great discrepancies between our finding and other studies results may be due to variation in foods, habits, cooking behaviors, and food keeping hygiene in addition to environmental factors such as the weather temperature and moist which significantly affect bacterial growth in food materials.

In this study, resistance gene (mecA) of *S*. *aureus* responsible for resistance to *β*-lactam antimicrobials was detected by using PCR, and we found that 15 (17%) *S*. *aureus* isolates were positive for the mecA gene while 69 (83%) were negative. Comparing our finding to previous studies results, we realized that most of the previous studies results showed a significantly higher prevalence than our finding, for example, in a study performed by Khayri in Makkah city, about 44.4% of his *S*. *aureus* isolates were positive for mecA gene [4]. Similarly, Papadopoulos and his colleagues found that 81.3% of their *S*. *aureus* isolates were positive for the mecA gene [3]. This variation could be due to the difference in the antibiotic protocol applied by doctors for their patients in different countries or due to the extensive usage of methicillin antibiotics by their communities or by doctors during treatment prescription in these countries that eventually lead to a high prevalence rate of MRSA. On the contrary, the results reported in this study are higher than those reported by Aydin et al. [5] where the mecA gene prevalence was only 11.4%. Low rates of mecA gene were also reported in Egypt, Brazil, and China where the prevalence of mecA gene was 5.1%, 9%, and 7.9%, respectively [6–8]. The variation between the results may be due to variation of samples sources and the use of different molecular techniques in different countries for the detection of mecA gene product.

In this study, one hundred raw meat samples were obtained from different supermarkets and restaurants in Khartoum State. These samples are examined for the presence of *S*. *aureus*. Thirty (30%) samples were found to be contaminated with *S*. *aureus*. These findings highlight the high potential risk for consumers of meat and dairy products especially in the absence of strict hygienic and preventive measures to avoid *Staphylococcus aureus* enterotoxins (SEs) production in foods. In other comparative studies, similar results were reported from the USA where the prevalence of *S*. *aureus* in meat samples was 29.0% [17]. Much lower results were reported in South Africa, who reported that S. *aureus* was 26.5% [18]. Also, our result is lower than that obtained by Das and Mazumder in India who reported that out of 65 samples, *S*. *aureus* incidence was in 46.1% [19]. In our study, none of the meat *S*. *aureus* isolates was resistant to ciprofloxacin and 13.3% was resistant to gentamicin. In disagreement with our result, Das and Mazumder in India found that 16.66% of S. *aureus* meat isolates were resistant to ciprofloxacin [19] and Pu et al. in Louisiana found that 13.0% was resistant to ciprofloxacin. Also, in contrast to our findings, Pu et al. in Louisiana found that 3.0% was resistant to gentamicin [20]. Vancomycin-resistant *Staphylococcus aureus* (VRSA) is a type of antibiotic-resistant *S*. *aureus* which have developed resistance and can no longer be treated with vancomycin. This study showed that 16.6% of the meat isolates were resistant to vancomycin, which suggests that the contamination may be coming from VRSA carrier's food handlers and processors; however, Das and Mazumder found that 3.33% of the isolates were resistant to vancomycin (VRSA) which is low compared with our findings [19]. Methicillin-resistant *S*. *aureus* (MRSA) strains have acquired a gene that makes them resistant to nearly all beta-lactam antibiotics; animal-adapted MRSA strains also exist. Although it is in small percentage, it has clinical importance and may cause serious problems to immunocompromised individuals as well as healthy ones (carriers). In this study, 6.6% of meat isolates were resistant to oxacillin; this finding was high compared to the results of van Loo et al. who found that 2.5% of *S*. *aureus* meat isolates were resistant to oxacillin [21] and low compared to the results of Das and Mazumder who found that 23.3% of *S*. *aureus* isolates were resistant to oxacillin [19].

Investigations through PCR technique on enterotoxin genes in this study showed the absence of enterotoxin genes in meat *S*. *aureus* isolates. A similar study in Denmark demonstrated the presence of enterotoxin genes in only 0.2% of the isolates [22]. On the other hand, a report from China demonstrated the prevalence of 46.0% enterotoxin gene [23], and Bergdoll found that the percentage of enterotoxigenic strains of *S*. *aureus* is estimated to be around 25% [24]. Moreover, most of *S*. *aureus* food isolates are not SEs producers; thus, a considerable research effort is still required for a better understanding of the interactions between *S*. *aureus* and the food matrix and of the mechanism of SEs production in foods stuffs [25]. The data obtained in this study probably underestimated the enterotoxigenic properties of the analyzed strains, since the possible presence of newly described SEs was not considered and the sample size was too small to represent *S*. *aureus*-contaminated meat effectively. However, there is always the possibility of mutation at the level of the corresponding gene, leading to the absence of its detection. Therefore, a positive PCR shows the presence of the enterotoxin genes, but a negative PCR does not point to the absence of the corresponding operon because there are many types of enterotoxin genes and we determine only one type [25].

In this study, one hundred white cheese samples collected from different retailers in Khartoum State to detect the rate of *S*. *aureus* contamination in cheese sample in this study is higher than that reported in Iran and Japan where the reported contamination rates were 16% and 13.3%, respectively [26, 27] and lower than that reported in Turkey which was 37.5% [28]. However, the results in this study disagree with a previous report in Khartoum State where no *Staphylococcus aureus* was found on white cheese [29]. This may be due to variation in sample sources where the cheese was manufactured, the retailers where cheese samples were purchased from, the sampling area, season, and environment; all these factors might affect the rate of contamination with microorganisms. The rate of sensitivity of cheese *S*. *aureus* isolates to gentamicin and ciprofloxacin was 100%; these findings oppose those reported in the USA, where 75% of *S*. *aureus* isolates were resistant to gentamicin [30] and in Iraq where 25% of *S*. *aureus* isolates were resistant to ciprofloxacin [31]. The resistance of cheese *S*. *aureus* to oxacillin in this study was 5%, which was lower than that reported in Iraq which was 20% [32]. The sensitivity to vancomycin was 85% which is lower than that reported in Switzerland which was 100% [33].

The molecular detection of *Staphylococcus aureus* enterotoxins (A, B, C, D, and E) genes among cheese isolates resulted in the detection of seb gene in 10% of the 20 isolates that was lower than the results obtained by Salheen in Sudan who reported that 20% of seb gene was detected in cheese samples [34]. The variation between results could be due to several factors such as sample source, geographical origin, the sensitivity of identification methods, and sample size which can affect the outcomes.

In this study, 108 fish samples were examined. The contamination rate of fish samples in this study was very low (22%) when compared with a previous report in Khartoum State (72%) [35], Egypt (93%) [11], and India (100%) [36] and relatively lower than that reported in Spain (27%) [37]. Antimicrobial susceptibility results for fish *S*. *aureus* isolates showed 100% sensitivity to ciprofloxacin, which was in agreement with reports in Egypt [11] and Turkey [38] and very close to a study and also nearly similar to reports in Portugal (98%) [39]. However, lower result in India was 48.5% [40] and in Egypt was 57% [41]. Gentamicin also showed an efficacy rate of 100% among all *S*. *aureus* isolates, which is similar to reports from Egypt [41], Turkey [38], and that of Vázquez-sánchez et al. in Spain [37] and slightly similar to results reported in Egypt (97%) [11] and in Portugal (92%) [39]. In this study, *S*. *aureus* isolated from fish samples showed a sensitivity rate of 92% to vancomycin, which is similar to those reported in Egypt (91%) [11] and Portugal (90%) [39], respectively. Another study in Egypt reported a higher result (100%) [41], while other studies in Turkey reported much lower sensitivity to vancomycin, which was 83% and 78%, respectively [38, 42]. The oxacillin showed 92% potency against fish *S*. *aureus* isolates which was higher than that reported in Portugal (62%) [39], while it was relatively lower than those reported in Spain and Turkey where both reported 100% [37, 38]. The variation between these results could be due to contribution of several factors such as source of samples, geographical origin, sensitivity of identification methods, and sample size which can affect the outcomes.

The molecular detection of the enterotoxin's genes among fish *S*. *aureus* isolates gave no band for all genes meaning that none of the all isolates possesses such gene in their genetic material; this finding agreed with those reported in Turkey and the USA [38, 43], where no enterotoxins B, C, and E genes were found in their food *S*. *aureus* isolates. However, a study in Tanzania reported that enterotoxins B and C genes were detected in 0.3% with the absence of enterotoxin A gene [44], while a study from Turkey reported enterotoxins A and D genes in 10.5% of their samples [38]. Variation among research studies' results and our findings could be due to several factors such as samples source, geographical origin, the sensitivity of identification methods, and sample size which can affect the outcomes.

In this study, *S*. *aureus* was isolated in 63% of raw milk samples, which is relatively close to that reported in Brazil (68%) [45] and Malaysia (60%) [46]. However, this finding was lower than those reported in Turkey (75%) [47] and Egypt (82%) [48]. The prevalence of this study is higher than those reported in two different studies in Iraq with prevalence of 53.33% and 43.5%, respectively [49, 50]. Moreover, the prevalence of *S*. *aureus* in this study is too high compared to reports from Sudan and Egypt where the levels were 30% and 24.8%, respectively [51, 52].*S*. *aureus* isolated from milk samples in this study were highly sensitive to ciprofloxacin (98.4%), which is close to a report from a study in Bangladesh (93.3%) [53]. However, relatively lower susceptibility levels were reported in India and Bangladesh where the rate was 80% and 83.3%, respectively [54, 55]. The sensitivity to gentamicin in this study (87%) is lower than that reported in Ethiopia (90%) [55, 56] and (100%) [57] and higher compared to another report in Sudan which was 47.6% [51].

While slightly similar to results obtained by Beyene in Ethiopia, Thaker et al. in India reported that 90% of *S*. *aureus* isolates were sensitive to gentamicin [55, 56]. Reports from other researchers indicated a higher level of susceptibility rate to gentamicin, for instance, Abraha et al. in Ethiopia reported that 100% of milk *S*. *aureus* isolates are susceptible to gentamicin [57]. In this study, the vancomycin susceptibility test was determined for all milk-originated *S*. *aureus*, and the result showed that 65% of the *S*. *aureus* isolates were susceptible to vancomycin; this finding was completely close to findings reported by Idbeis, in Basrha in Iraqe, and AL-Marsomy and Bendahou et al., in North Morocco, who mentioned that *S*. *aureus* isolated from raw milk and milk products showed sensitivity to vancomycin (100%) [58–60]. On the contrary, studies from Ethiopia and Iraq reported 100% resistance to vancomycin [57, 61]. The sensitivity to oxacillin (84%) in this study is higher than that reported in India (70%) [55] and lower than that reported in Bangladesh (100%) [54]. All milk *S*. *aureus* isolates tested for the presence of enterotoxins genes yielded negative results. Similar findings were reported in Hungary [62]. The variations between results reported in this study and other reports could be attributed to several factors such as samples source, geographical origin, sensitivity of identification methods, and sample size which can affect the outcomes.

## 5. Conclusion

There was a relatively moderate prevalence of methicillin-resistant *Staphylococcus aureus* with very low frequency of enterotoxin B gene in different kinds of selected food samples collected from Khartoum State. These findings elucidate the increased risk on public in Khartoum being affected by Staphylococcal food poisoning upon consumption of dairy or meat products prepared in unhygienic conditions that could lead to intoxication by *Staphylococcus aureus* enterotoxins.

## Figures and Tables

**Table 1:** Antimicrobial sensitivity pattern of *Staphylococcus aureus* isolated from different food material samples.

Isolates	Pattern	Antibiotics			
		Gentamicin (10 mg)	Ciprofloxacin (5 mg)	Oxacillin (5 mg)	Vancomycin (30 mg)
Meat	Sensitive	26 (86.7%)	30 (100%)	28 (93.4%)	25 (83.3%)
	Resistant	4 (13.3%)	0 (0%)	2 (6.6%)	5 (16.7%)

Cheese	Sensitive	20 (100%)	20 (100%)	19 (95%)	17 (85%)
	Resistant	0 (0%)	0 (0%)	1 (5%)	3 (15%)

Fish	Sensitive	24 (100%)	100 (100%)	22 (92%)	22 (92%)
	Resistant	0 (0%)	0 (0%)	2 (8%)	2 (8%)

Milk	Sensitive	55 (87.3%)	62 (98.4%)	53 (84%)	41 (65%)
	Resistant	8 (12.7%)	1 (1.6%)	10 (16%)	22 (35%)

Total		137	137	137	137

**Table 2:** Primers used for detection of *S. aureus* housekeeping gene, enterotoxins, and mecA genes.

Primer	Sequence 5'–3'	Product size (bp)
Housekeeping gene primers		
Staph 756-F	AACTCTGTTATTAGGGAAGAACA	—
Staph 750-R	CCACCTTCCTCCGGTTTGTCACC	756
Enterotoxins genes primers		
SA-Ua-F	TGTATGTATGGAGGTGTAAC	—
SA-A-R	ATTAACCGAAGGTTCTGT	270
SA-B-R	ATAGTGACGAGTTAGGTA	165
ENT-C-R	AAGTACATTTTGTAAGTTCC	102
SA-D-R	TTCGGGAAAATCACCCTTAA	303
SA-E-R	GCCAAAGCTGTCTGAG	213
mecA gene primers		
MecA1–F	AACTCTGTTATTAGGGAAGAACA	—
MecA1–R	CCACCTTCCTCCGGTTTGTCACC	310

Ua, universal; f, forward; r, reverse.

**Table 3:** Distribution of bacteria isolated from selected food samples purchased from retailers in Khartoum State.

Isolate	Number	Percentage (%)
*Staphylococcus aureus*	137	34.25
Others bacteria	126	31.5
No growth	137	34.25
Total	400	100

**Table 4:** Distribution of mecA and enterotoxin genes among *Staphylococcus aureus* isolates.

Type of gene detected	Positive	Negative	Total
mecA gene	15 (17.9%)	69 (82.1%)	84 (100%)
Enterotoxin B gene	2 (2.38%)	82 (97, 62%)	84 (100%)
Other enterotoxin genes	0 (0%)	84 (100%)	84 (100%)

**Table 5:** Distribution of *Staphylococcus aureus* isolates according to the type of food samples.

Sample	*S*. *aureus* isolates	Other bacteria isolates	No growth	Total
Meat	30 (30%)	20 (20%)	50 (50%)	100 (100%)
Cheese	20 (20%)	4 (4%)	76 (76%)	100 (100%)
Fish	24 (24%)	76 (76%)	0 (0%)	100 (100%)
Milk	63 (63%)	26 (26%)	11 (11%)	100 (100%)
Total	137	126	137	400

## Data Availability

The datasets analyzed during the current study are available from the corresponding author upon request.
